# Elevated axonal membrane permeability and its correlation with motor deficits in an animal model of multiple sclerosis

**DOI:** 10.1186/s40035-017-0075-7

**Published:** 2017-02-28

**Authors:** Gary Leung, Melissa Tully, Jonathan Tang, Shengxi Wu, Riyi Shi

**Affiliations:** 1 Department of Basic Medical Sciences, College of Veterinary Medicine, Purdue University, West Lafayette, IN 47907 USA; 2 Weldon School of Biomedical Engineering, Purdue University, West Lafayette, IN 47907 USA; 3 MSTP program, Indiana University School of Medicine, Indianapolis, IN USA; 4 Department of Neurobiology, Fourth Military Medical University, Xi’an, China

**Keywords:** Multiple sclerosis, EAE, Axonal membrane damage, Polyethylene glycol, Acrolein, Horseradish Peroxidase, Membrane permeability, Neurodegeneration

## Abstract

**Background:**

It is increasingly clear that in addition to myelin disruption, axonal degeneration may also represent a key pathology in multiple sclerosis (MS). Hence, elucidating the mechanisms of axonal degeneration may not only enhance our understanding of the overall MS pathology, but also elucidate additional therapeutic targets. The objective of this study is assess the degree of axonal membrane disruption and its significance in motor deficits in EAE mice.

**Methods:**

Experimental Autoimmune Encephalomyelitis was induced in mice by subcutaneous injection of myelin oligodendrocyte glycoprotein/complete Freud’s adjuvant emulsion, followed by two intraperitoneal injections of pertussis toxin. Behavioral assessment was performed using a 5-point scale. Horseradish Peroxidase Exclusion test was used to quantify the disruption of axonal membrane. Polyethylene glycol was prepared as a 30% (w/v) solution in phosphate buffered saline and injected intraperitoneally.

**Results:**

We have found evidence of axonal membrane disruption in EAE mice when symptoms peak and to a lesser degree, in the pre-symptomatic stage of EAE mice. Furthermore, polyethylene glycol (PEG), a known membrane fusogen, significantly reduces axonal membrane disruption in EAE mice. Such PEG-mediated membrane repair was accompanied by significant amelioration of behavioral deficits, including a delay in the emergence of motor deficits, a delay of the emergence of peak symptom, and a reduction in the severity of peak symptom.

**Conclusions:**

The current study is the first indication that axonal membrane disruption may be an important part of the pathology in EAE mice and may underlies behavioral deficits. Our study also presents the initial observation that PEG may be a therapeutic agent that can repair axolemma, arrest axonal degeneration and reduce motor deficits in EAE mice.

## Background

Although inflammation is known to be the major pathology of multiple sclerosis (MS), the mechanisms underlying tissue damage and functional loss remain unclear [1, 2]. While myelin degeneration has long been considered the primary neuropathological characteristic for MS, recent studies indicate that axonal degeneration is also an important component of the pathology [3–5]. In fact, there is strong evidence suggesting that MS is a neurodegenerative diseases [3, 6–8]. Indeed, the integrities of both myelin and axons are indispensable for neuronal function and survival [9]. Therefore, either myelin or axonal damage could theoretically lead to axonal conduction loss and degeneration seen in MS [10–13]. Consistent with this notion, it has been suggested that axonal disruption may represent irreversible neurodegeneration in patients with MS [3]. This may in part explain why conventional strategies focusing solely on myelin protection have resulted in few effective treatments to slow or prevent MS progression [1].

Despite its potential importance in MS, axonal damage has attracted significantly less attention compared to myelin damage while both are known to lead to neurodegeneration in MS [6, 7, 14]. Consequently, the pathological role of axonal damage in MS remains insufficiently characterized. Specifically, the key cellular processes that trigger axonal degeneration remain unclear. We have previously shown that axonal membrane damage contributes to axonal degeneration observed in CNS trauma [11–13, 15–17]. We have also shown that acrolein, a pro-inflammatory aldehyde that is capable of inflicting axonal membrane damage and functional loss [18–24], is elevated and likely plays an important pathological role in MS [25]. In light of this evidence, we speculate that damage to the axonal membrane, or axolemma, likely leads to neuronal degeneration and loss of neurological function, and therefore contributes to the development and progression of symptoms observed in MS.

Polyethylene glycol (PEG), a hydrophilic polymer, is well known for its ability to seal neuronal membranes and consequently restore integrity and associated neuronal function [13, 26–29]. In particular, it has been shown that PEG is capable of repairing axolemmal damage and provide neuroprotection in traumatic spinal cord injury [19, 26–28, 30–40]. However, the therapeutic effect of PEG has not been examined in non-traumatic CNS illnesses, such as MS, in which axonal membrane damage likely plays a role leading to axonal degeneration. Therefore, the primary focus of this study was to determine whether axolemmal disruption can be detected and to examine its possible correlation with functional deficits associated with MS. Subsequently, we also aimed to confirm the pathological role of axolemmal disruption in MS and to assess the therapeutic efficacy of administering PEG as a membrane sealant.

## Methods

### Experimental autoimmune encephalomyelitis mice

We certify that all applicable institutional and governmental regulations concerning the ethical use of animals were followed during the course of this research. Female C57BL/6 mice were purchased from Harlan Laboratories (Indianapolis, IN, USA) and were housed in the Purdue University veterinary animal housing facilities. Ten to twelve week old mice received two subcutaneous injections of 0.1 mL myelin oligodendrocyte glycoprotein/complete Freud’s adjuvant emulsion (EK-0115, Hooke Laboratories, Lawrence, MA, USA) into the upper and lower back. Immediately following the emulsion injections, 0.1 mL of pertussis toxin (EK-0115, Hook Laboratories) was administered intraperitoneally to the mice, and again 22–26 h later. Behavioral assessment was performed using a 5-point scale [41]. Animals were placed on a metal grate to record their walking ability and motor function. The behavioral scale used was as follows: 0 – no deficit; 1 – limp tail only; 2 – hind limb paresis without frank leg dragging; 3 – partial hind limb weakness with one or both legs dragging; 4 – complete hind limb paralysis; 5 – moribund, paralysis in hind limbs and forelimbs. These studies were approved by the Purdue Animal Care and Use Committee, Purdue University, West Lafayette, IN.

### Horseradish peroxidase exclusion test

**Fig. 1:**
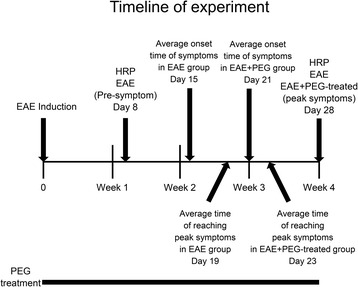
Experimental design and time course. Diagram illustrates the time course of the overall experiment related to EAE, pre-symptom (EAE) and peak symptom (both EAE and EAE + PEG-treated) groups. In addition, the time points when the HRP-exclusion assay was conducted are illustrated: at pre-symptom (day 8 post-induction) and at peak symptom (Day 28 post-induction), with or without PEG treatment. Behavioral analysis was conducted daily and the average time of onset of symptoms, and the average time of reaching peak symptoms for both the EAE and the EAE + PEG group are illustrated. In the EAE + PEG group, PEG treatment was carried out for the entire period of study starting on the day of induction, as indicated in the diagram. Tissue samples for HRP testing were extracted for 4 different groups: healthy controls, pre-symptom EAE mice, peak symptom EAE mice and PEG-treated EAE mice

The mice were separated into 4 groups: healthy control mice, EAE mice before the onset of symptoms (pre-symptom), EAE mice at peak behavioral deficit (peak symptoms), and PEG-treated EAE mice. After confirmation of behavior at various pre-determined experimental end points (Fig. 1), each group of animals was anesthetized with Ketamine (90 mg/kg) and Xylazine (10 mg/kg) and perfused (intra-cardiac) with a cold, oxygenated Krebs solution. The spinal columns were quickly removed from the animal and a complete laminectomy was performed. The spinal cord was then excised from the vertebrae and placed in cold, oxygenated Krebs solution containing 0.015% horseradish peroxidase (Sigma Type IV, Sigma Aldrich) for 2 h. The tissue was then fixed in 2.5% glutaraldehyde in phosphate buffer for 4 h at room temperature. After fixation, a Vibratome (Electron Microscopy Science, Hatfield, PA, USA) was used to cut 30 μm transverse sections of the tissue. Tissue was then processed in a diaminobenzidene solution to visualize HRP uptake by damaged. Digital images of HRP-stained spinal cord sections were obtained with an optical microscope connected to a computer. Stained axons were counted and expressed as density (axons/mm^2^) using Image J analysis [15, 17, 30]. Animals were sacrificed for structural analysis at pre-induction (control), 8 days post-induction (pre-symptom), or 4 weeks post-induction (peak symptom).

### Polyethylene glycol treatment

Polyethylene glycol (295906, Sigma Aldrich, St. Louis, MO, USA) was prepared as a 30% (w/v) solution in phosphate buffered saline. The solution was then filtered for sterilization and injected into each animal every day after induction for the whole study (4 weeks post-induction). A volume of 0.1 mL was administered intraperitoneally daily after induction for the duration of the study. EAE only animals were administered the same amount of saline rather than PEG.

### Statistical analysis

Throughout the paper, Mann Whitney *U* test was used to compare the difference of the severity of motor defects, the onset of motor deficits, and the onset of peak symptoms between EAE and EAE + PEG-treated groups. Kruskal-Wallis test was used for comparison of membrane permeability assessed by HRP-exclusion test in various groups. The statistical significance level was set at *p* < 0.05. All data are expressed as mean ± standard error of the mean (SEM).

## Results

### Significant axonal membrane damage in EAE mouse and its reduction by Polyethylene glycol

**Fig. 2:**
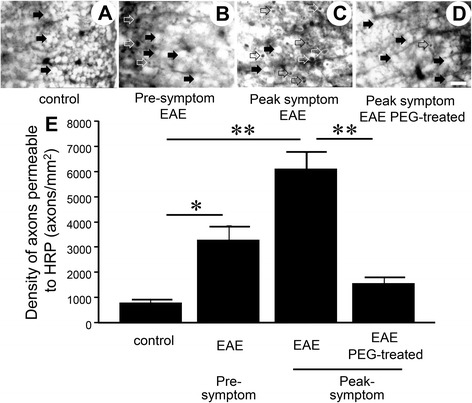
Membrane permeability assessed by HRP-exclusion test. Horseradish peroxidase (HRP)-exclusion test determined amount of axonal membrane damage in healthy control mice (*n* = 5), pre-symptom EAE mice (*n* = 7), peak symptom EAE mice (*n* = 6), and peak symptom EAE + PEG-treated mice (*n* = 7). For pre-symptom EAE group, samples were taken at 8 days post-induction, before symptom emerges. The samples of peak symptom group (for both EAE and EAE + PEG-treated) were taken at 28 days post-induction. **a**–**d** The images represent HRP-stained sections of spinal cord tissue from the four groups. *Solid arrows* denote areas in which HRP did not penetrate the cell while the *open arrows* point to areas depicting HRP penetration revealing increased axonal membrane permeability. **e** The *bar graph* quantifies HRP uptake in each group. The average density for the control group was 811 ± 130 axons/mm^2^. The peak symptom group had the highest levels of axonal damage (6147 ± 665 axons/mm^2^, ^**^ *P* < 0.01 compared to control) while the pre-symptom group exhibited increased levels compared to the control group (3293 ± 500 axons/mm^2^, ^*^ *P* < 0.05 compared to control). In addition, HRP labeling in the EAE/PEG-treated group (1581 ± 247 axons/mm^2^) is significantly lower than EAE group (^**^ *p* < 0.01). Scale bar = 10 mm for **a**, **b**, **c** and **d**

Using an EAE mouse model, we first examined axonal membrane damage using a well-established HRP-exclusion assay. The integrity of axonal membrane from the spinal cord of control mice, EAE mice before the onset of symptoms (pre-symptom), and EAE mice at peak behavioral deficit (peak symptom) was examined (Fig. 1). We have found that the average HRP labeling for these conditions was 811 ± 130 axons/mm^2^, 3293 ± 500 axons/mm^2^, and 6147 ± 655 axons/mm^2^ respectively (Fig. 2). EAE mice at peak deficit demonstrated significantly higher levels of HRP labeling compared to control mice (*P* < 0.01). Interestingly, pre-symptom EAE mice also displayed increased axonal membrane permeability compared to control mice (*P* < 0.05).

We further examined the possibility that PEG can reduce axonal membrane permeability. Specifically, EAE mice were given daily injections of 0.1 mL of either polyethylene glycol (30% w/v) (EAE + PEG), or saline (EAE only) beginning on the first day of induction and then daily for 4 weeks (Fig. 1). Axonal integrity based on the HRP exclusion assay was carried out at the end of the treatment period. We have found that the density of HRP-labeled axons in the EAE-PEG treated group was 1581 ± 247 axons/mm^2^, which is significantly lower than that of the EAE only mice at peak deficit (6147 ± 655 axons/mm^2^, *p* < 0.01) (Fig. 2).

### Polyethylene glycol temporally ameliorates motor deficits in EAE mice

**Fig. 3:**
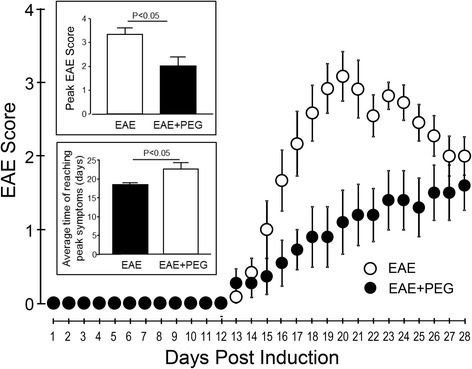
PEG ameliorated the severity of motor defects and delayed the time of reaching peak motor deficits. Comparison of behavioral assessment each day between EAE (saline-treated, *n* = 12) and EAE + PEG (EAE-treated with PEG, *n* = 11) groups. The graph represents the average score for each group of animals throughout the study. The *upper inset* demonstrates that administration of PEG significantly decreased the peak symptom severity in EAE mice (*P* < 0.05). The highest score of each animal was recorded and averaged within each group to quantify the mean score of severity to be used for the *upper inset*. As indicated, the average of the peak EAE score in EAE + PEG group (1.91 ± 0.4) is significantly lower than that in EAE group (3.33 ± 0.3, *P* < 0.05). The *lower inset* describes the average time of reaching peak symptom of motor deficits in both EAE and EAE + PEG groups. For both EAE and EAE-PEG group, the time of reaching peak symptom is defined as the time that the most severe symptom, or the highest EAE score (≥2) first appears for each animal. If an EAE score of 2 is never reached during the experimental duration (28 days), then 28 day is used as the time of reaching peak symptom. As shown, the average time of reaching the peak EAE score in EAE + PEG group (23.1 ± 1.6) is significantly longer than that in EAE group (18.7 ± 0.8, *P* < 0.05)

In addition to axonal membrane permeability examination, we also carried out behavioral analysis in two experimental groups, EAE and EAE + PEG mice. The behavioral observation for each animal was recorded daily on a 5-point scale immediately following induction and continuously for 4 weeks. The average behavioral score was calculated for each day in two groups and displayed over time in Fig. 3. The severity of behavioral deficit in the EAE/PEG-treated group was significantly lower than the EAE group during the period of day 16 to 25 days post EAE induction. When averaging the highest scores of each animal within each group, the PEG-treated EAE mice (1.91 ± 0.4) displayed a significantly lower score than the EAE mice (3.33 ± 0.3, *P* < 0.05) (Fig. 3 upper inset). In addition, PEG treatment also significantly delayed the time of reaching peak symptoms (23.1 ± 1.6 days for EAE-PEG, and 18.7 ± 0.8 days for EAE group, *P* < 0.05) (Fig. 3 lower inset).

**Fig. 4:**
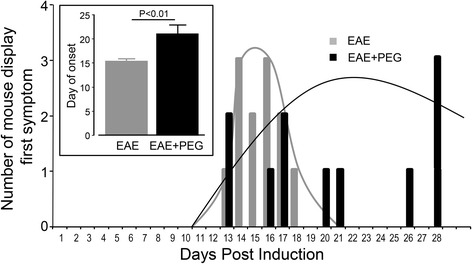
PEG delayed the onset of motor deficits. Comparison of onset of symptoms between EAE (saline-treated, *n* = 12) and EAE + PEG (EAE-treated with PEG, *n* = 11) groups. The graph represents the temporal distribution or symptom onset for EAE and EAE + PEG mice. The number of mice showing initial symptoms was recorded daily for each group. Symptom onset for EAE mice was tightly clustered early in the study while onset for EAE + PEG mice was more dispersed. The *inset graph* represents the average day of onset for EAE and EAE + PEG groups. Animals in EAE + PEG group developed symptoms significantly later in the study compared to EAE mice (*P* < 0.01). Three mice in the EAE + PEG-group never developed symptoms and were counted as day 28 for both graphs

In addition to decreased peak symptom severity and delayed time to reach the peak symptom, PEG treated animals also showed delayed symptom onset as depicted in Fig. 4. Specifically, in the EAE group, all mice began to display their behavioral deficit between days 13 and 18. In contrast, the EAE + PEG group revealed a more dispersed result with a trend of delayed onset. Specifically, five EAE + PEG animals began exhibiting symptoms at approximately the same time as the EAE group (13–18 days induction) while the others were later in time: three exhibited no observable behavioral deficit throughout the 4-week observation (counted as day 28 when averaging) while the remaining three mice showed their first motor defects at between day 20–26 post induction. Overall, the average day of onset for EAE mice that received PEG-treatment was 20.63 ± 1.8 days which is significantly delayed compared to EAE mice (15.42 ± 0.4 days, *P* < 0.01) (Fig. 4).

## Discussion

Based on the current study using the HRP-exclusion assay, a well-established method of assessing axonal membrane permeability [11, 13, 15–17, 30, 42], we have determined that there is a significant level of axonal membrane disruption in EAE mice when symptoms peak. In addition, we also noted a prominent increase, although at a lesser degree, of axonal membrane damage in the pre-symptom stage in EAE mice (Fig. 2). To our knowledge, this is the first evidence of axonal membrane damage in EAE mice, an animal model MS.

Furthermore, PEG, a known membrane repairing agent [28–31], when applied daily for 4 weeks after induction, can significantly reduce axonal membrane disruption in EAE mice (Fig. 2). The restoration of axonal membrane integrity by PEG was accompanied by significant amelioration of behavioral deficits, including a delay of the onset of motor deficits typical of EAE, a delay of emergence of peak symptom, and a reduction of peak symptom severity (Figs. 3 and 4). Taken together, we have presented initial evidence that axonal membrane disruption is an important feature of the overall pathology in EAE mice that is at least in part responsible for the behavioral deficits. Our study also presents the first indication that PEG could be used as a therapeutic agent to effectively repair axolemma, arrest axonal degeneration and reduce motor deficits in EAE mice.

Although not examined in this study, it is likely that PEG-mediated axonal membrane repair also leads to the reduction of axonal degeneration, a known pathology of MS [3, 4, 43]. It is well known that axonal membrane disruption, if not repaired, will lead to axonal degeneration, neuronal cell death and overall neuronal tissue degeneration [13, 17, 44–46]. It has also been demonstrated repeatedly that PEG-mediated neuronal membrane repair can lead to the reduction of oxidative stress and mitochondrial dysfunction which are known contributors to axonal degeneration and neuronal cell death [19, 26, 29, 35, 47]. As such, PEG may also provide neuroprotection by indirectly suppressing oxidative stress and inflammation. Therefore, we postulate that PEG-mediated membrane repair can mitigate axonal degeneration and could promote a range of cellular functions that lead to the improvement of motor function in EAE mice.

Although we did not confirm the presence of PEG inside the spinal cord in the current study following systemic application, we believe the main location of PEG treatment is in the central nervous system (CNS), particularly in spinal cord. This is because we have detected significant membrane repair in spinal cord when PEG was applied comparing to no PEG (Fig. 2), and PEG is known to be able to reach spinal cord following systemic application [48]. It is unlikely that PEG-mediated neuroprotection is due to peripheral effects, considering the main pathology of EAE is in CNS [2].

Despite the strong evidence of severe axonal membrane damage in EAE, the mechanisms of such axolemmal damage remain to be elucidated. Based on the previous studies from our and other labs, we suggest that acrolein, a lipid peroxidation byproduct, may be one of the culprits. In a recent study from our lab it was shown that acrolein was increased significantly in EAE mice [25]. We have also shown that acrolein can cause membrane disruption in various preparations at levels that are likely achievable in in vivo pathological conditions [20–22, 49–51]. In fact, acrolein has been suggested to cause neuronal damage in trauma by disrupting neuronal membrane through a delayed mechanism [24, 49, 50, 52–54]. In light of this evidence, we hypothesize that acrolein may play a role in axonal membrane disruption in EAE mice. One critical piece of evidence supports this hypothesis is that hydralazine, an effective acrolein scavenger, can lower acrolein levels and reduce motor deficits in EAE mice [25]. In addition, a recent study from our group demonstrated that acrolein-mediated axonal conduction loss can be partially mitigated by a potassium channel blocker, indicating a concomitant acrolein-mediated myelin damage in addition to axonal lesions [24]. This is because augmented potassium channel activity is a known consequence of myelin damage in injured axons [55]. Consistent with such notion, we also have found that acrolein trapping treatment was associated with restoration of neuronal membrane integrity, reduced neurodegeneration and enhanced functional recovery in traumatic spinal cord injury [21, 22, 24, 50, 51, 53]. It will be interesting to confirm the likely scenario that anti-acrolein therapy alone could lead to the preservation or restoration of axonal membrane integrity in EAE.

In the current study, in addition to the severe membrane disruption observed when symptoms peak, we also noted a less severe, yet still significant level of membrane disruption, and therefore neurodegeneration, in the pre-symptom period defined as a week prior to the emergence of motor deficits (Figs. 1 and 2). Therefore, significant level of membrane damage and neurodegeneration appear to already exist in the pre-symptom period while no concomitant noticeable behavioral deficits were detected. This phenomenon may be explained by the fact that there is a significant amount of implicit redundancy of axons to support neuronal function. Therefore, there is likely a threshold level of axonal damage and neurodegeneration that must be reached before observing behavioral changes. This would justify the notion that membrane damage could start before the onset of behavioral deficits and that the emergence of behavioral deficits signifies a critical level of axonal damage and degeneration. Hence, the initial membrane damage could theoretically serve as an indication to predict the onset of behavioral deficits at a later date.

In light of these observations related to the relationship between axonal membrane damage and behavioral performance, we suggest that axonal membrane damage in MS could serve as an important diagnostic measurement. First, it can potentially be used as a biomarker for the detection of MS before the emergence of symptoms. This could help to institute earlier treatments to deter neurodegeneration for better therapeutic outcomes provided that the pre-symptomatic axonal membrane damage can be firmly identified. Second, axonal membrane integrity could also serve as an indication of the effectiveness of membrane repair therapy such as PEG. Such a strategy is understandably dependent on development of a reliable non-invasive imaging method to detect axonal membrane disruption or degeneration.

It has become increasingly clear that, in addition to myelin damage, axonal degeneration may also play a critical role in EAE pathology. From a basic cellular biology view, the integrity of both myelin and the axonal membrane are essential for axonal conduction. Therefore, axonal degeneration along with myelin damage, are likely to be equally important contributing factors in axonal conduction failure and behavioral deficits in EAE mice [5, 12, 15, 17, 54, 56, 57]. The current study further highlighted the critical role of axonal damage, and axolemmal disruption in particular, in neurodegeneration and the functional loss in MS. To further stress the importance of axonal degeneration in MS, there is evidence that axonal damage and neurodegeneration may be the main cause of functional loss without obvious myelin damage in some human case of MS. For example, based on a histopathologic investigation using autopsy tissue from MS patients, Trapp and his colleagues have shown compelling evidence of axonal degeneration in the absence of myelin loss [43]. Therefore, axonal damage, a key feature of neurodegeneration, may not just be one of the important compounding pathological factors, but rather it may be among the primary and critical factors that are sufficient to cause clinical functional deficits in MS. To further highlight the importance and causal role of axonal damage in MS pathology, we have noted a significantly higher susceptibility of acrolein-mediated axonal damage compared to acrolein-mediated myelin damage. Specifically, Shi and his colleagues have found that using an ex vivo preparation of extracted rodent spinal cord segment, the threshold of acrolein needed to inflict membrane damage is two magnitudes lower than that needed to cause significant myelin damage [10, 49]. This suggests that in MS patients, axonal degeneration may be, in some cases, the primary pathology that precedes myelin damage. Furthermore, due to the existing evidence of damaging both axon and myelin, acrolein may be a critical link for the damage of both myelin and axons, two known pathologies in EAE. This hypothesis is supported by the factor that acrolein scavenging could mitigate the damage of both myelin and axons [25, 26, 29].

The emergence of the importance of axonal damage as the critical pathology warrants closer examination of our existing therapeutic strategies as well as our efforts to establish new therapies. It appears reasonable to suggest that a treatment regimen should include axonal repair and protection in addition to myelin protection. This combination of treatments may be a synergistic treatment strategy and could result in increased efficacy. We have previously shown that both anti-acrolein (by hydralazine) and membrane repair (by PEG), when used alone, can offer significant, albeit partial alleviation of behavioral deficits [25] (Fig. 3). Furthermore, though PEG significantly delayed the onset and reduced severity in most of the symptomatic periods, such symptom reduction was temporary. PEG treatment did not lead to significant behavioral improvement beyond 26 days post induction, despite effective sealing of the axonal membrane. Therefore, it appears that PEG-mediated membrane repair alone cannot offer long-term symptom reduction. This could be the case for at least two reasons. First, this may indicate that membrane repair strategies need to be combined with approaches that remove causes of cellular damage, such as acrolein, to ultimately protect the cell. Second, although a proven axonal membrane repair agent, PEG has not been shown to also repair myelin damage.

## Conclusions

Our findings demonstrate that there is significant axonal membrane damage in addition to myelin destruction and both likley contribute to neurodegeneration in EAE mice. Further, the impairment of axon and myelin may require distinct protective measures and both are critical for a normalization of neuronal function. Taken together, PEG-mediated membrane repair strategy may need to be combined with other measures designed to protect and repair myelin in order to maximize the therapeutic effect and ultimate functional preservation and recovery in MS.

## Abbreviations

EAE: Experimental autoimmune encephalomyelitis
MS: Multiple sclerosis
PEG: Polyethylene glycol
